# A human behavior-based model for respiratory infectious diseases prediction

**DOI:** 10.3389/fpubh.2025.1578178

**Published:** 2025-04-29

**Authors:** Zhengwen Ma, Min Zhu, Chen Zhi, Huaguo Zhang, Minye Li, Nan Zhang, Hui Ma

**Affiliations:** ^1^School of Nursing, Southern Medical University, Guangzhou, China; ^2^Department of Infection Control, Sixth Medical Center, PLA General Hospital, Beijing, China; ^3^Nursing Department, PLA General Hospital, Beijing, China; ^4^Anding Hospital, Capital Medical University, Beijing, China; ^5^Faculty of Urban Construction, Beijing University of Technology, Beijing, China

**Keywords:** respiratory infectious diseases, relative distance, relative facial orientation, relative position, model, behavior

## Abstract

**Objectives:**

The research aims to develop a human behavior-based model to predict respiratory infectious diseases.

**Methods:**

This research employs semi-supervised machine learning techniques in conjunction with an RGB-depth camera to collect micro-level data. We employed computational fluid dynamics to simulate the dispersion of virus concentration in outpatient environments. Furthermore, we evaluated the infection risk of respiratory infectious diseases (RIDs) by utilizing a dose–response model.

**Results:**

A total of 201,600 behavioral data points were collected. The average interpersonal distance observed during medical procedures was 0.62 meters. The most common facial orientation between patients and healthcare workers (HCWs) was face-to-face, accounting for 30.48% of interactions. The predicted average viral RNA load exposures per second during various medical procedures were as follows: Otoscopy: 0.014314 viral RNA loads/s; Rhinoscopy: 0.014411 viral RNA loads/s; Laryngoscopy: 0.014379 viral RNA loads/s; External auditory canal irrigation: 0.018803 viral RNA loads/s. Simulations of preventive measures indicated that N95 masks reduced the probability of infection to 2.44%, surgical masks to 14.81%, and cotton masks to 36.05%.

**Conclusion:**

This research presents an innovative micro-level exposure risk model for respiratory infectious diseases (RIDs), which provides significant insights into the risk of infection. However, it is important to acknowledge certain limitations, including the distinctiveness of the data sources utilized and the insufficient examination of transmission pathways. Subsequent studies should aim to enhance the dataset, fine-tune model parameters, and integrate further transmission pathways to augment both the accuracy and applicability of the model.

## Introduction

The recent surge in respiratory infectious diseases (RIDs) has emerged as a significant threat to public health security (1). The World Health Organization (WHO) estimates that the COVID-19 pandemic has resulted in over 760 million confirmed cases and approximately 6 million deaths worldwide (2). Although global efforts have temporarily mitigated the spread of COVID-19, RIDs continue to pose a substantial risk to human health (3). Current research suggests that RIDs may reduce global life expectancy by an average of 1.29 years (4). Therefore, it is imperative to enhance awareness and implement preventive and control measures in both daily life and workplace settings to effectively address the ongoing challenges (5) presented by COVID-19 and the potential resurgence of other respiratory viral diseases (6).

Recent studies have suggested the application of predictive models, including the Susceptible-Infected (SI) Model (7), the Susceptible-Infected-Recovered (SIR) Model (8), and the Seasonal SIR Model (SIRS) (9), to effectively forecast RIDs. These models utilize historical data to estimate the probability of infection; however, their reliance on past occurrences, such as the number of susceptible and infected individuals, limits their efficacy in predicting the emergence of new respiratory infectious diseases (10). Therefore, rather than serving as robust forecasting tools, these models primarily illustrate trends during RIDs outbreaks. In addition to these models, other methodologies such as neural networks (11), spatially weighted Poisson regression (12), seasonal decomposition of time series (13), Long Short-Term Memory (LSTM) networks (14), and Autoregressive Integrated Moving Average (ARIMA) models (15) have been employed to predict epidemiological patterns of RIDs. While these models have significantly contributed to the field of mathematical epidemiology, they often necessitate the continuous updating of data or the utilization of extensive datasets to derive optimized system parameters. Furthermore, they may fail to explain the critical issue of social interactions among individuals (16). As a result, macroscopic models can exhibit insensitivity in assessing the dynamics of infectious diseases or require stringent assumptions to mitigate their inherent limitations. Conversely, microscopic models offer a partial remedy to these issues by concentrating on the transmission of disease at the individual level (17). However, employing microscopic models to describe the mechanisms of virus transmission still presents challenges (18). As a result, the formulation of accurate microscopic models holds significant importance (19). The collection of behavioral data has emerged as a significant barrier to advancing micro-level modeling research. Although Sorokowska (20) employed survey methods to investigate interpersonal distance and Hajime Kanamori (21) gathered data on human touch behavior through direct observations, both approaches are susceptible to biases; surveys may suffer from memory inaccuracies, while on-site observations are inevitably influenced by observer error. Therefore, there is a pressing need for innovative technological solutions to facilitate the acquisition of more accurate and reliable behavioral data, overcoming the limitations of traditional research methodologies.

This study aimed to employ a novel technique that integrated RGB depth cameras with semi-supervised machine learning algorithms to collect behavioral data. The infection risk evaluation for RIDs was enhanced through the development of micro-level transmission models, which utilized the human behavioral dataset to quantify exposure.

## Methods

### Study design and setting

This study employed a hybrid computational-experimental methodology that integrated computational fluid dynamics (CFD) simulations with behavioral observation data. The research was conducted in an outpatient department, focusing specifically on healthcare workers exposure dynamics.

### Environment and equipment preparation

**Figure 1:**
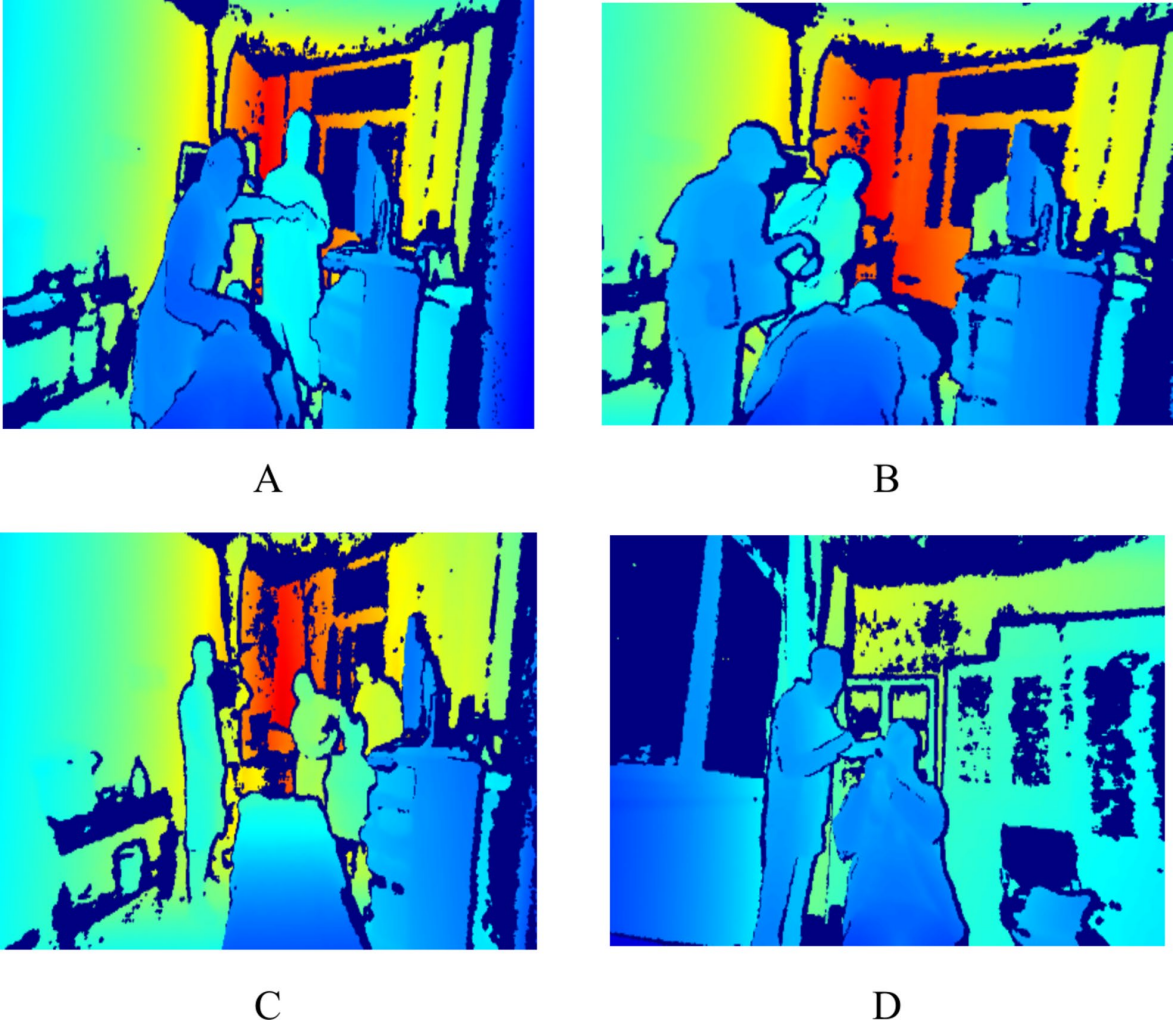
Sample of behavioral data (**A** indicates nasal endoscopy, **B** indicates laryngeal endoscopy, **C** indicates otoscopy, and **D** indicates auditory canal irrigation).

The research was conducted at a comprehensive, first-rate hospital in Beijing, China. The otolaryngology department of the outpatient clinic was selected as the primary research site to ensure the site’s representativeness. The tools utilized in the study included a tripod, a portable hard drive, a portable power supply, a depth camera, and a portable computer. The portable computer was equipped with PyCharm Community Edition software and a Linux operating system, with pre-edited code imported into the project. This code encompassed the Kinect_RGBD_Cap project, which records behavioral data; the labeling4RGBD project, which generates label annotations for the data; and the Yolov_8 project, which employs semi-supervised machine learning techniques on the data. The Kinect_RGBD_Cap project is capable of capturing three-dimensional information of the environment and measuring the distance between the target and the camera, thereby generating depth maps. The specific methodology for data collection is as follows: first, the user should launch the PyCharm Community Edition software and initiate the Kinect_RGBD_Cap project to capture behavioral data, as illustrated in Figure 1. Subsequently, the data should be saved to the designated working folder to complete the data collection process. The Yolov8 algorithm represents a machine learning approach that integrates a limited amount of labeled data with a substantial volume of unlabeled data for model training. Its significance lies in facilitating the rapid collection and processing of behavioral data for this research.

### Data collection

Based on observations conducted during the pre-experimental phase, the observation area was positioned within 6 meters of the medical operation area. This proximity was established to enhance the accuracy and reliability of the behavioral data collected. During the research period, data were continuously collected over a duration of 7 days, with collection occurring from 7:30 to 12:00 and from 13:00 to 17:00 each day. To maximize the simulation of real-world behaviors, a total of 201,600 pieces of behavioral data were monitored. This dataset includes pertinent medical procedures such as otoscopy, nasal endoscopy, laryngeal endoscopy, and auditory canal irrigation. The privacy of patients and healthcare professionals was safeguarded, as the images captured were depth images that did not contain identifiable facial features; please refer to Figure 1. This research received ethical approval from the Ethics Committee of the People’s Liberation Army General Hospital of China (S2023-522-01).

### Data cleaning

In the collection of data on human behavior, two trained investigators meticulously examine the data. Based on prior research, we have established the following filtering criteria: (1) The original behavioral data cannot determine the relative distance between healthcare personnel and patients; (2) the original behavioral data cannot clearly identify the relative facial orientation between healthcare personnel and patients; (3) the original behavioral data cannot locate the relative position of healthcare personnel and patients. If the original data exhibiting one or more of these characteristics will be excluded from this study. Following this filtering process, 161,917 valid human behavior data points remain from the original 201,600 data points. The excluded data are all invalid data, and the proportion of excluded data does not exceed 20%, thereby further ensuring the reliability of the data.

### Annotated data

**Figure 2:**
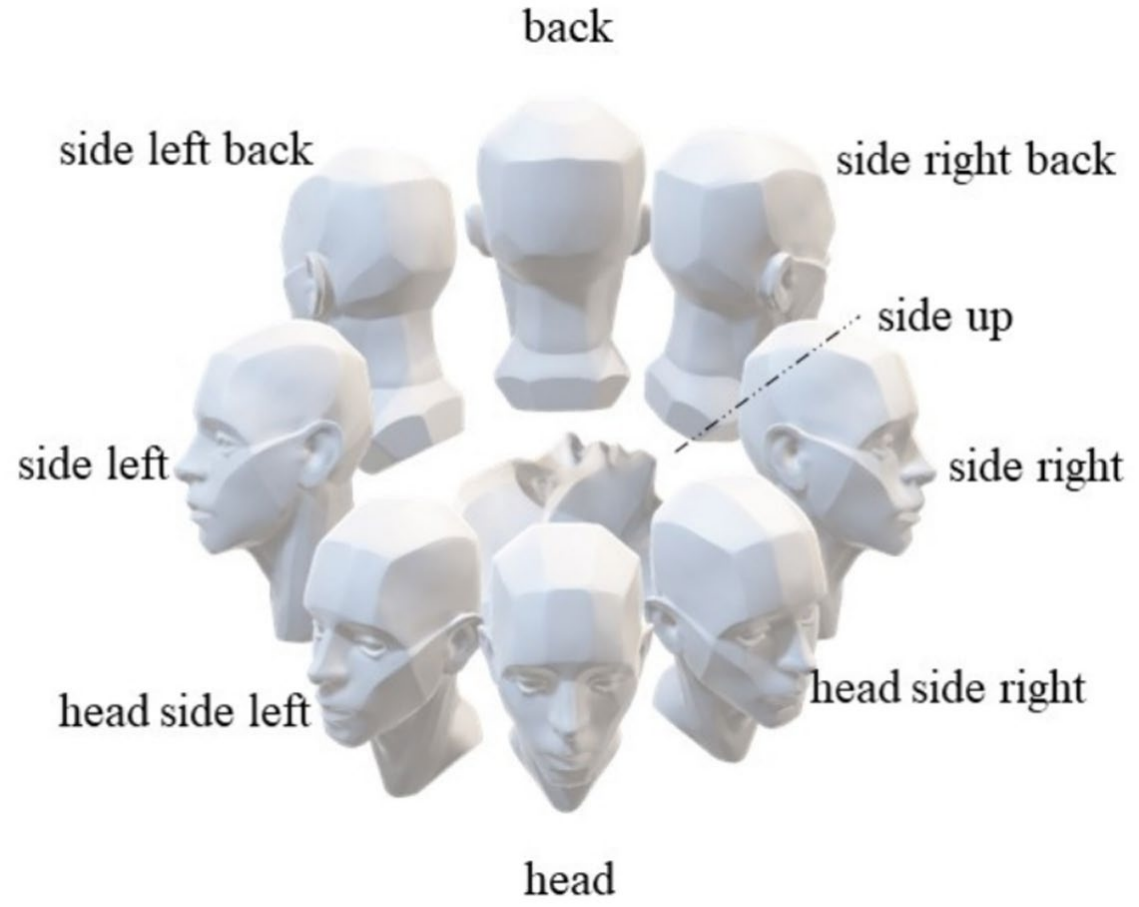
The orientation of the individual’s face.

We have developed face orientation labels based on the participants’ relative positions to the depth camera, aiming to identify the relative facial orientations among participants to achieve more accurate human behavior identification. As illustrated in Figure 2, these labels include “front,” “back,” “right side,” “left side,” “upward,” “right front side,” “left front side,” “right back side,” and “left back side.”

### Training behavioral data

The YOLOv8 model was utilized in this study to conduct semi-supervised machine learning on behavioral data. We selected the following metrics to objectively assess the model’s performance:

a) Precision: As demonstrated in Equation 1, it quantifies the percentage of actual positive cases among those that the model predicted to be positive.
b) Recall: As demonstrated in Equation 2, recall quantifies the percentage of all actual positive occurrences that the model accurately predicts as positive.
c) Mean Average Precision: As illustrated in Equation 3, this metric calculates the model’s average performance across all categories.

(1) $$\mathrm{Precision}=\frac{TP}{TP+FP}$$

(2) $$\mathrm{Recall}=\frac{TP}{TP+FN}$$

(3) $$\mathrm{mAP}=\frac{1}{c}\overset{c}{\underset{i=1}{\sum }}AP_{i}$$

TP stands for True Positives, FP stands for False Positives, FN stands for False Negatives, and C represents the number of classes in the dataset. The higher the AP value, the better the performance of the model.

### Developing a close-range transmission model for SARS-CoV-2

This study exclusively focused on the close-range transmission of SARS-CoV-2 within outpatient settings, excluding surface transmission. As a result, the research evaluated the infection risk faced by healthcare workers and investigated the mechanisms of pathogen transmission occurring at close range in an outpatient context. The initial parameter settings were based on the following assumptions:

1) The five relative orientations identified from the facial parameters between healthcare workers (HCWs) and patients, based on facial direction analysis utilizing machine vision technology, are Face-to-Face (F-F), Face-to-Side (F-S), Face-to-Back (F-B), Side-to-Side (S-S), and Back-to-Back (B-B).
2) In the indoor environment of the clinic, we hypothesized that susceptible individuals acquired the virus through two primary mechanisms: direct inhalation and indirect deposition. Simultaneously, infected individuals disseminated the virus within the environment through verbal communication and respiration.
3) Aerosols serve as the primary vector for virus transmission. However, as exhalation patterns have evolved, so too has the particle size distribution of aerosols. Aerosols with a particle size of less than 5 μm are classified as small, while those larger than 5 μm are classified as large. Recent studies indicate that all particles expelled during regular breathing are aerosols with a small particle size (22). Both large and small aerosols are released during speaking (23). Consequently, the six identified transmission categories are: small particle-size aerosol inhalation during breathing, small particle-size aerosol sedimentation during breathing, small particle-size aerosol inhalation during speaking, small particle-size aerosol sedimentation during speaking, large particle-size aerosol inhalation during speaking, and large particle-size aerosol sedimentation during speaking.
4) In this study, we incorporated experimental results obtained from computational fluid dynamics (CFD). The attenuation coefficients associated with variations in facial orientation, referred to as *η*(f) in this manuscript, were derived from simulations examining inhalation and sedimentation viral exposure for both large and small aerosol particle sizes across various facial orientations. Furthermore, to ascertain the attenuation coefficients pertinent to changes in distance, also designated as η(d) in this paper, the experiments simulated inhalation and sedimentation viral exposure for large and small aerosol particle sizes at relative distances of 0.3 m, 0.5 m, 0.7 m, 0.9 m, 1.1 m, 1.3 m, 1.5 m, and 2.5 m.
5) The generation rates of small particle-size aerosols released during breathing, small particle-size droplets released during speaking, and large particle-size aerosols released during speaking were determined in this study through a review of literature. The rates were found to be 1.73 × 10^−8^ μL/s, 1.95 × 10^−8^ μL/s, and 2.39 × 10^−3^ μL/s, respectively (24).
6) Viruses were generated at a rate of 4.5 × 10^-2 viral RNA copies per second in small particle-size aerosols produced by breathing, 1.0 viral RNA copies per second in small particle-size aerosols produced by speaking, and 7.4 × 10^-2 viral RNA copies per second in large particle-size aerosols produced by speaking (23).
7) We reevaluated the virus exposure levels based on the demonstrated protective efficacy of N95 (25), surgical, and fabric masks (26). This approach provides a more comprehensive assessment of their protective capacities, allowing for a better evaluation of the effectiveness of various preventative measures.
8) The following model was employed in this study to illustrate the close-range transmission of SARS-CoV-2:

(4) $$e\left(s,r\right)=vG\left(R\right)\cdot \eta \left(d\right)\cdot \eta \left(f\right)\cdot C\left(s\right)$$

In Equation 4, e(s, r) denotes the level of viral exposure, quantified as viral RNA load per second (viral RNA load/s), where s represents the particle size and r reflects the various exhalation patterns, including inhalation and deposition. The function vG(R) indicates the volume flow rate of aerosols, measured in microliters per second (μL/s), corresponding to a particle size of s, which is generated by the exhalation activities R, such as speaking or breathing, of the infected individual. Additionally, the *η*(d) refers to the attenuation coefficient associated with relative distance, while η(f) represents the attenuation coefficient related to relative facial orientation. Lastly, C signifies the concentration of the virus, expressed as viral RNA load per second (viral RNA load/s), within aerosols of particle size s.

## Results

### Analysis of personnel behavior

**Figure 3:**
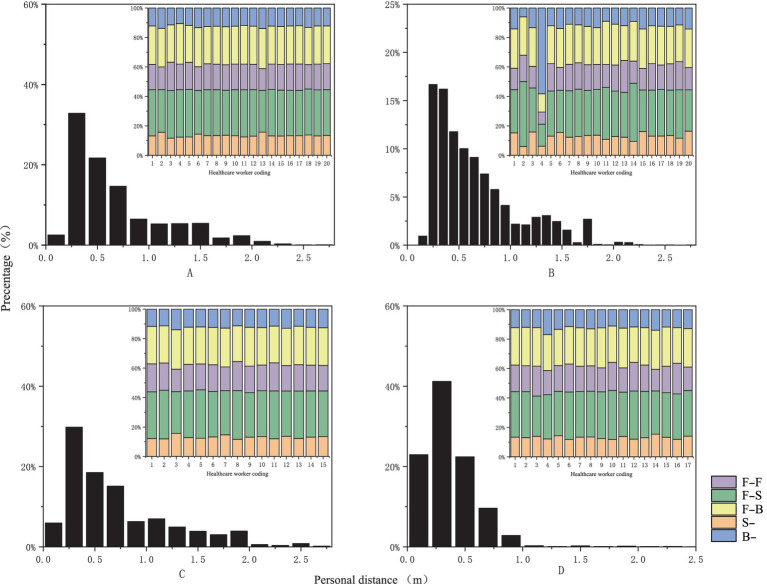
Probability distribution graph of interpersonal distance and facial orientation [the horizontal axis represents relative distance and healthcare provider codes, while the vertical axis indicates frequency distribution, measured in percentage. **(A)** Represents nasal endoscopy, **(B)** indicates laryngeal endoscopy, **(C)** denotes otoscopy, and **(D)** signifies auditory canal irrigation].

We collected 201,600 data points by monitoring behavioral interactions in the outpatient otorhinolaryngology department. The findings revealed a high percentage of close-range interactions (90.62%) and an average interpersonal distance of 0.62 meters between HCWs and patients (see Table 1). The primary facial orientations observed among HCWs and patients were F-F, F-S, and F-B, accounting for 17.1, 30.5, and 25.2% of the total, respectively (see Figure 3).

**Table 1:** Interpersonal distance between HCWs and patients.

Medical operation	Total	Mean	Standard	Sum	Min	Median	Max
A	20	0.67508	0.22163	13.50164	0.42709	0.61215	1.1213
B	25	0.70838	0.20353	17.70946	0.3996	0.63334	1.19879
C	15	0.69932	0.21287	10.48976	0.48231	0.5984	1.1077
D	27	0.3915	0.07712	6.65548	0.27598	0.3703	0.58176

### Training results for the model

**Figure 4:**
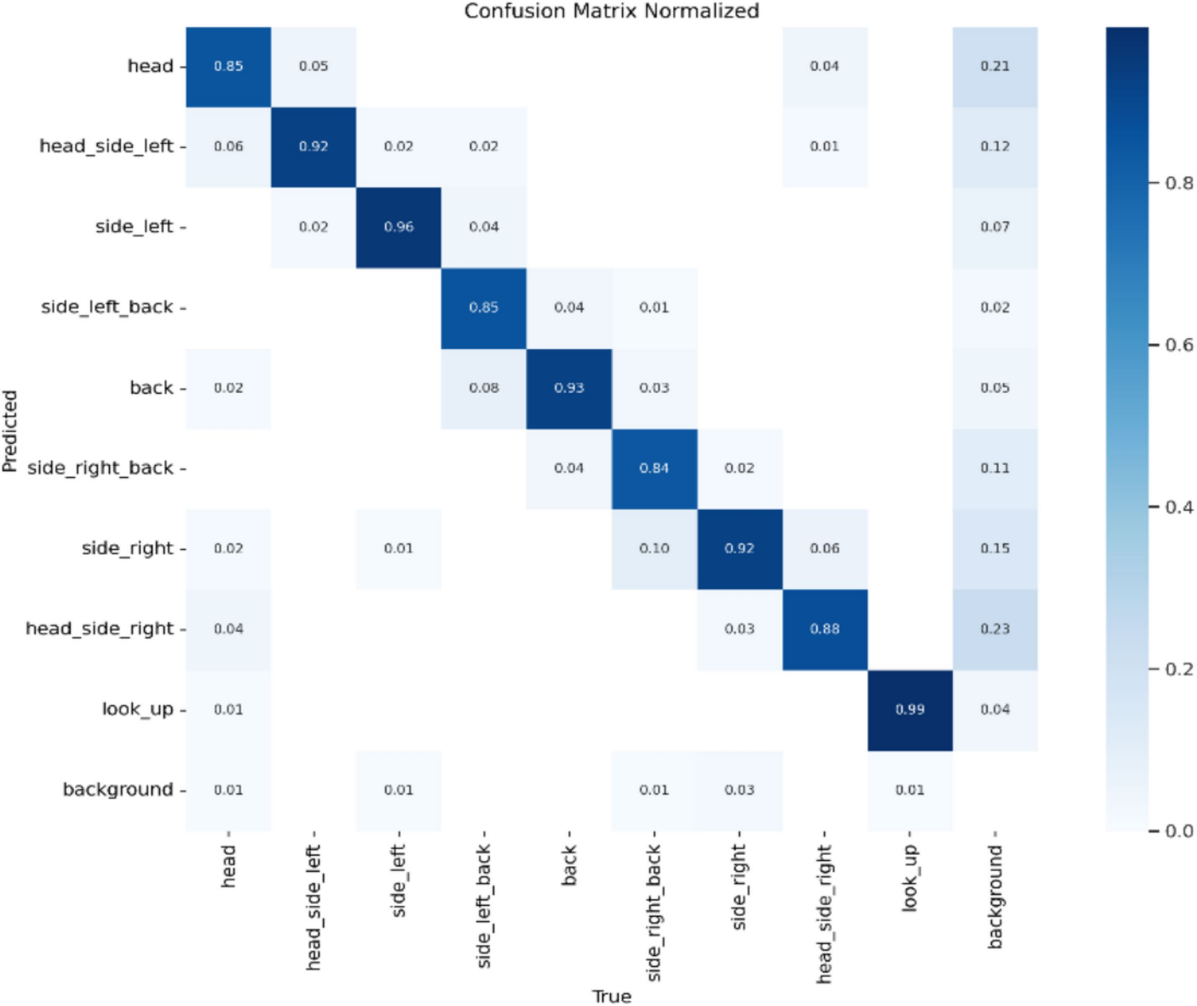
Standardized confusion matrix.

Model Accuracy: The results of this study’s model are summarized using a standardized confusion matrix. The confusion matrix clearly illustrates whether the model misclassifies one category as another or confuses two distinct categories. As shown in Figure 4, the actual categories are represented by the columns (x) in the matrix, while the predicted categories are represented by the rows (y). The average accuracy rate is 90.4%, indicating that the model effectively recognizes and classifies data.

**Figure 5:**
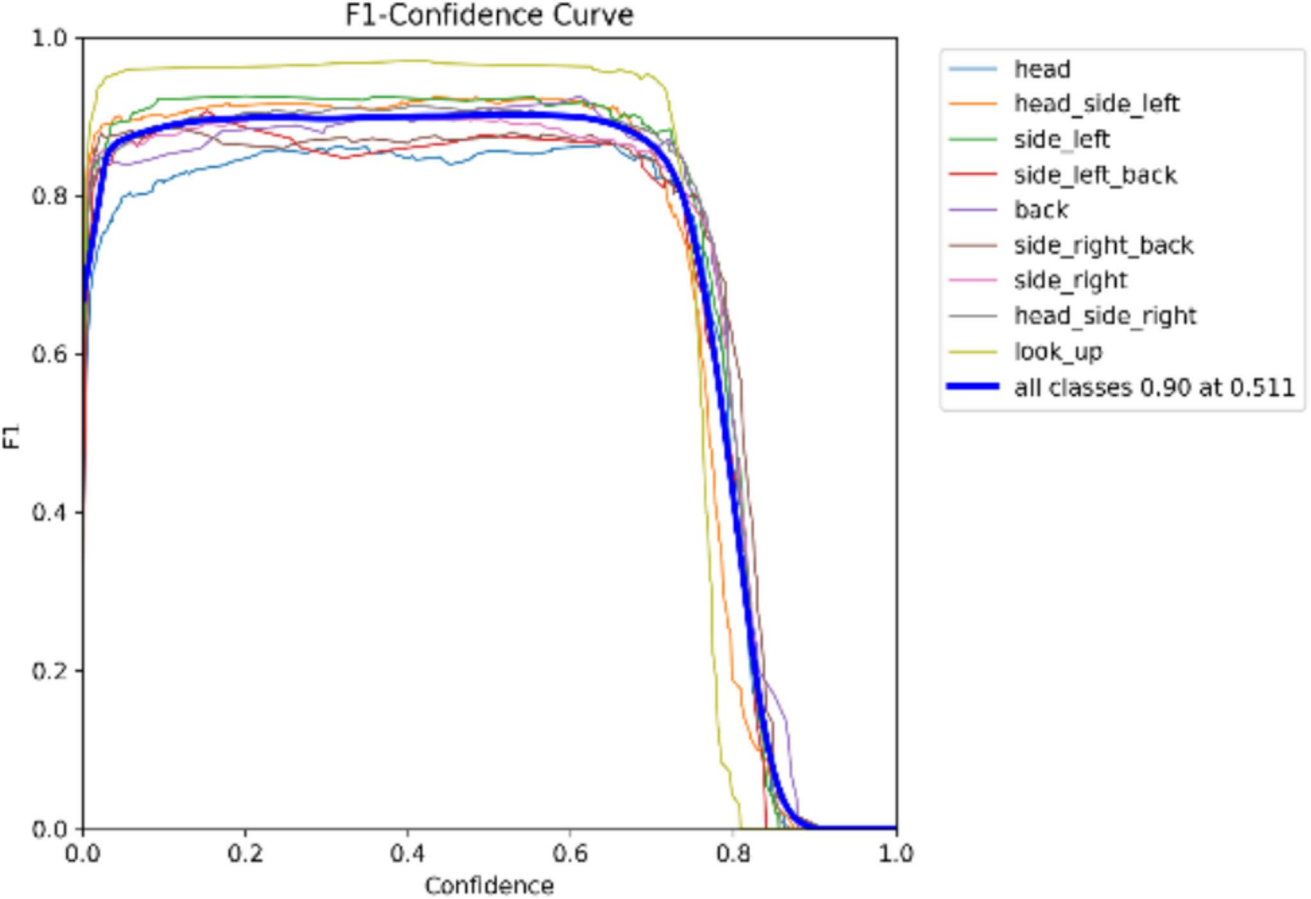
F1 curve.

**Figure 6:**
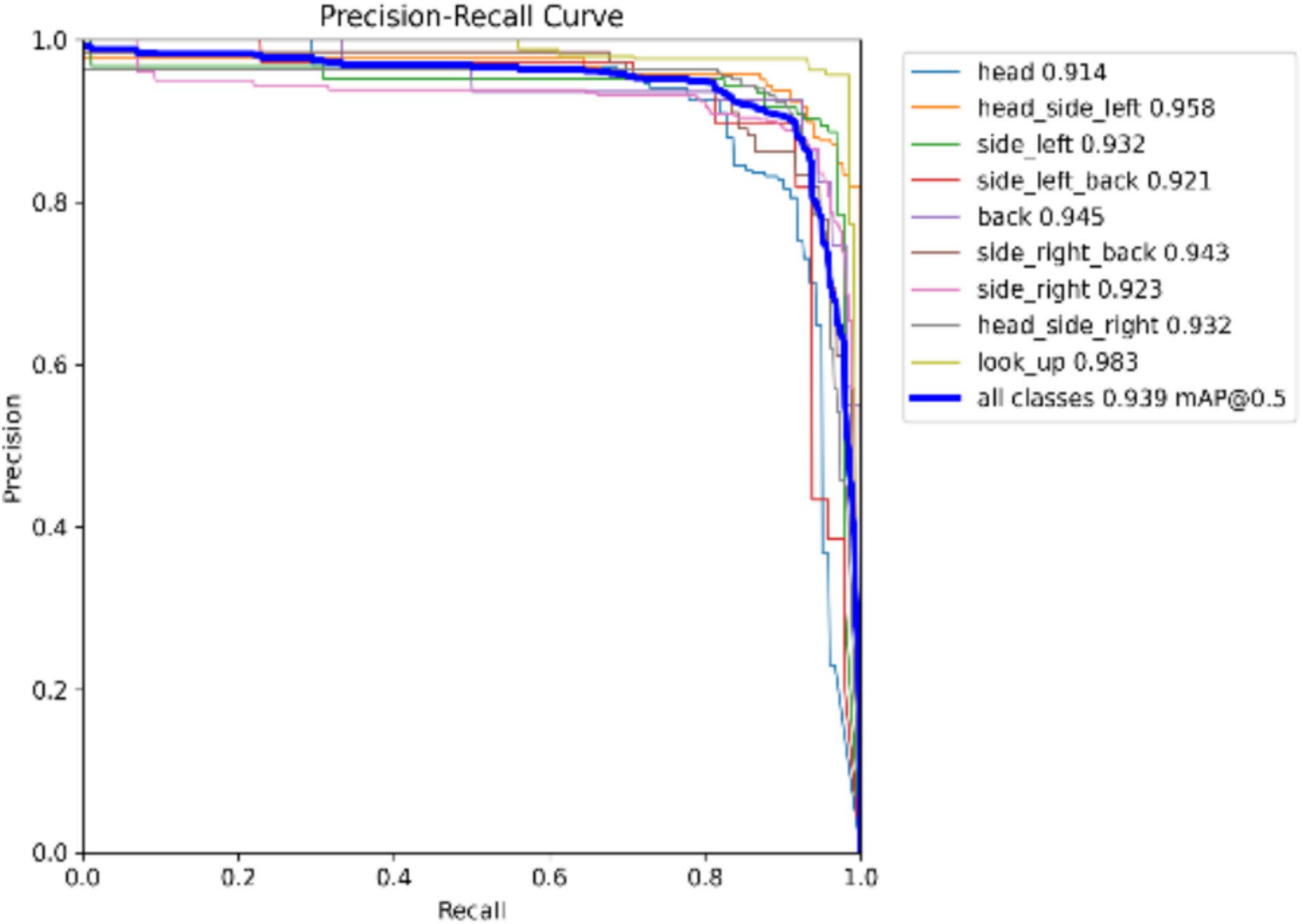
P_R curve.

Model Precision: This study employs precision and recall to assess the overall accuracy of the model. The F1 score, which is derived from the harmonic mean of precision and recall, ranges from 0 to 1, with 1 indicating optimal performance and 0 indicating the worst performance. Ideally, an F1 score within the confidence interval of 0.4 to 0.6 suggests that the model performs adequately. The Precision-Recall (PR) curve illustrates the relationship between precision and recall, with recall plotted on the horizontal axis and precision on the vertical axis. Typically, as recall increases, precision decreases, and vice versa. However, this study finds that the closer the curve is to the upper right corner, the better the model’s ability to accurately predict outcomes while maintaining high precision and recall, as shown in Figures 5, 6.

**Figure 7:**
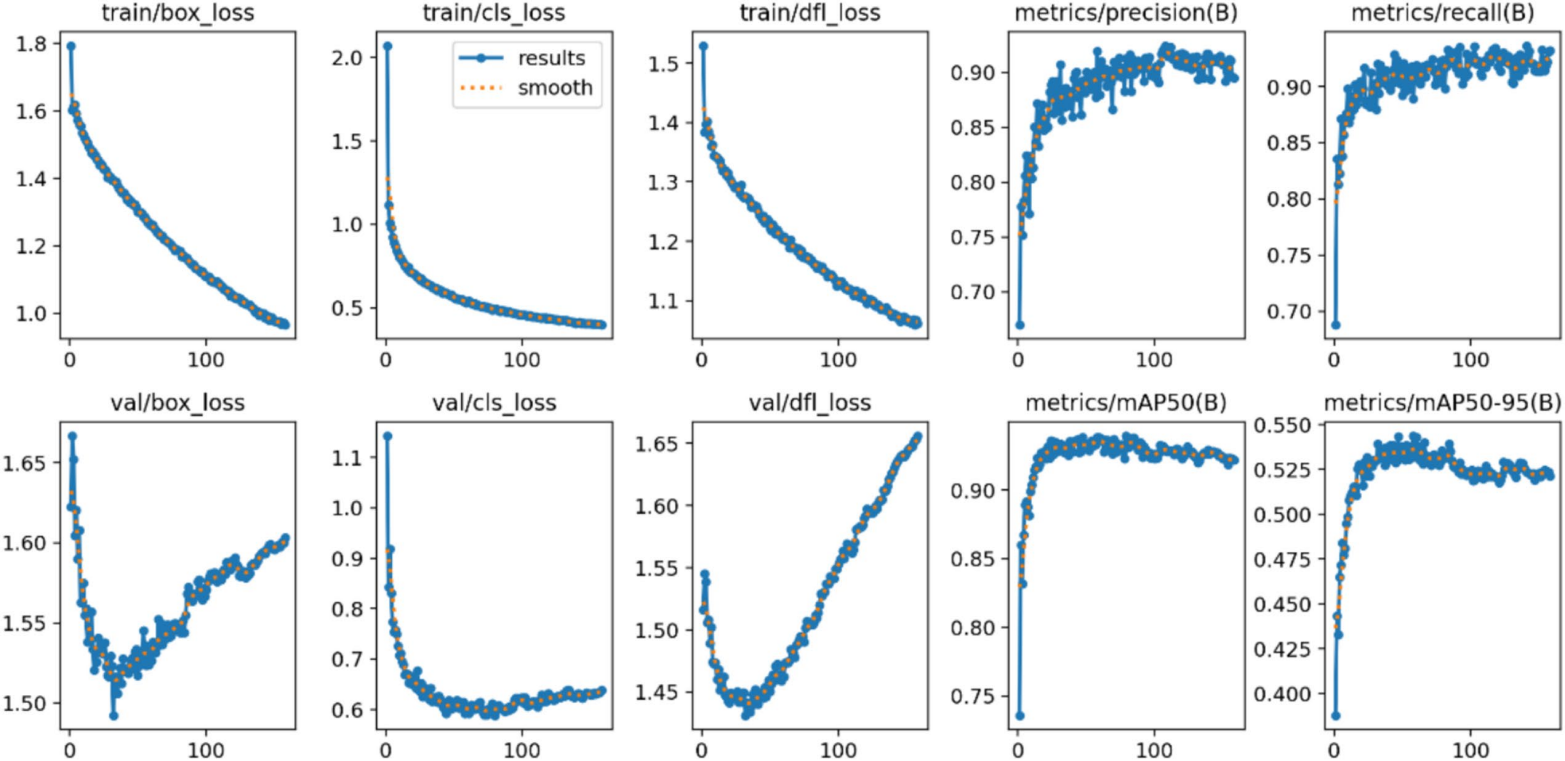
Average precision of the model.

Average Precision of the Model: The localization loss is represented by the Box loss, where a smaller value indicates that the model can locate the target more accurately. The classification loss is represented by the Cls loss, where a smaller value signifies that the model can more accurately identify different categories. The confidence loss is represented by the dfl loss, which enables the model to accurately determine whether the target is present. As illustrated in Figure 4, a smaller value corresponds to better performance. Based on the combination of these parameters and the findings from the mean Average Precision (mAP) output, it is reasonable to conclude that the model possesses strong recognition capabilities and can be effectively utilized for rapid batch identification of human behavior data (Figure 7).

### Viral exposure levels

**Figure 8:**
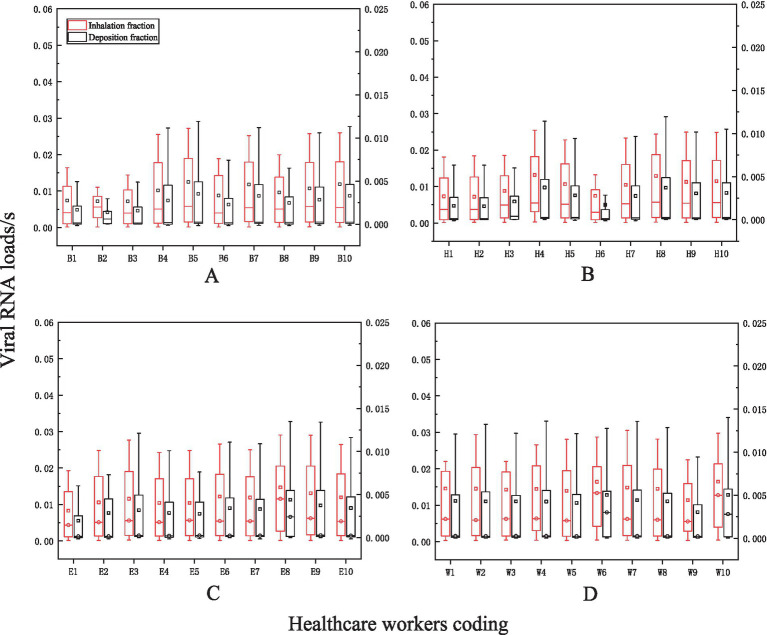
The levels of viral exposure per second. The vertical axis represents the viral exposure level per second (unit: “viral RNA load/s the horizontal axis indicates the coding for healthcare workers (HCWs). Nasal endoscopy is labeled as **(A)**, laryngeal endoscopy as **(B)**, otoscopy as **(C)**, and auditory canal irrigation as **(D)**.

An average of 0.0113 viral RNA loads/s through inhalation and 0.0031 viral RNA loads/s through deposition during nasal endoscopy. At laryngeal endoscopy, the average viral exposure level was 0.0113 viral RNA loads/s through inhalation and 0.00308 viral RNA loads/s through deposition. During otoscopic examination, the average viral exposure level was 0.01123 viral RNA loads/s through inhalation and 0.003 viral RNA loads/s through deposition. During auditory canal irrigation, the average viral dose per second via the inhalation route was 0.0145 viral RNA loads/s, while the viral exposure via the deposition route was 0.0043 viral RNA loads/s (Figure 8).

### Viral exposure levels and human behavior

**Figure 9:**
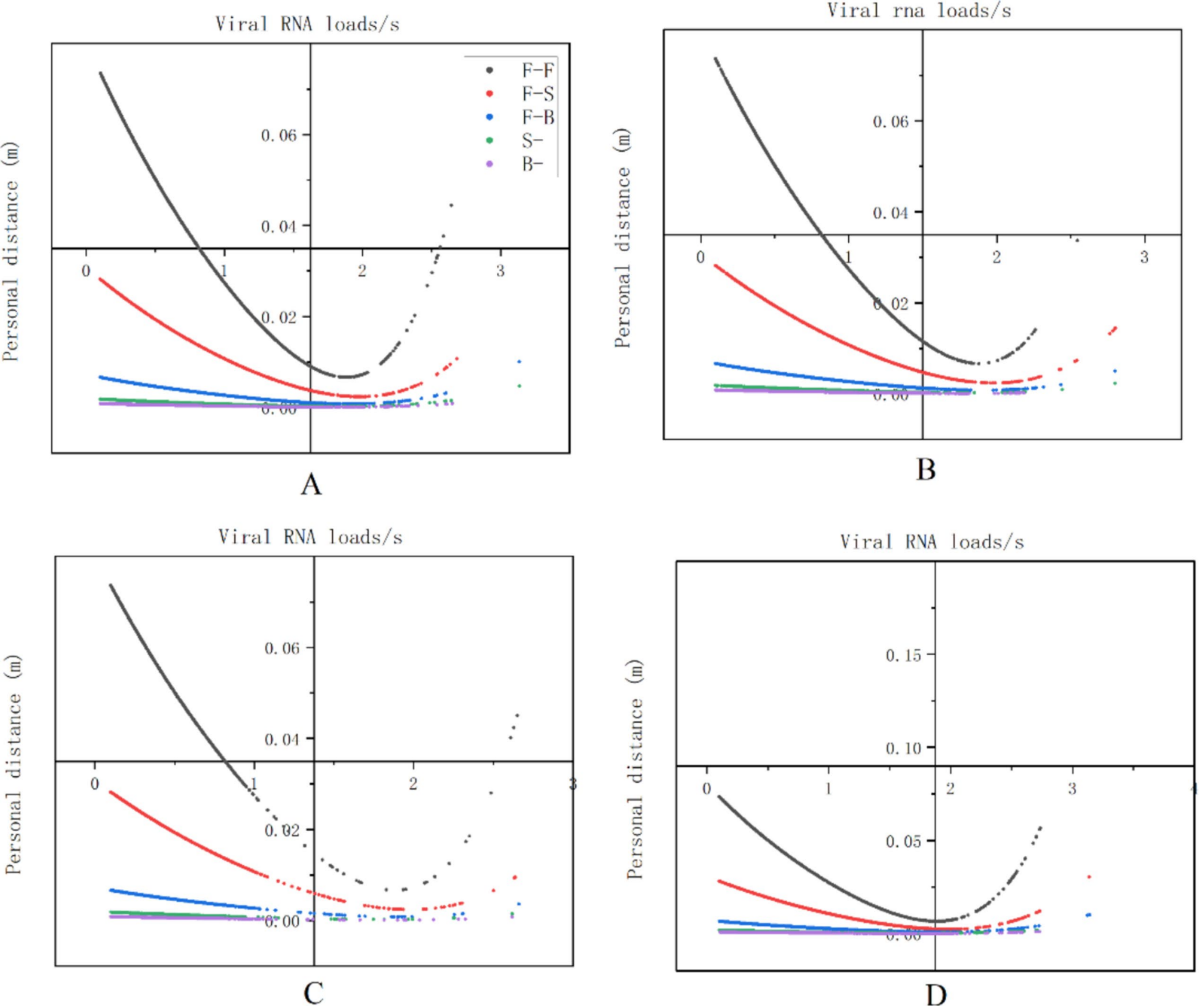
The relationship between viral exposure levels and human behavior. The vertical axis represents viral exposure levels, measured in “viral RNA load/s,” while the horizontal axis indicates interpersonal distance, measured in meters (m). The labels **(A–D)** correspond to nasal endoscopy, laryngeal endoscopy, otoscopy, and auditory canal irrigation, respectively.

Additionally, our findings demonstrated that the average level of viral exposure per second was highest during nasal endoscopy, laryngeal endoscopy, otoscopy, and auditory canal irrigation when the relative facial orientation between HCWs and patients were positioned face-to-face (F-F) position. This was followed by the face-to-side (F-S) orientation, with the lowest exposure occurring during the back-to-back (B-B) facial orientation. Furthermore, as interpersonal distance increased, levels of viral exposure decreased (see Figure 9).

### Intervention strategies’ efficacy

**Figure 10:**
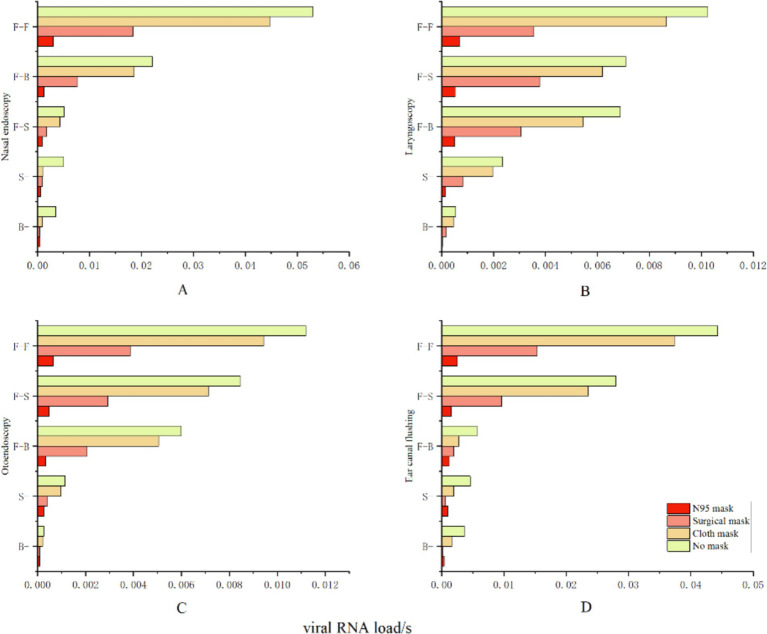
Protective effectiveness of various intervention measures. The vertical axis represents relative facial orientation, with **(A)** denoting nasal endoscopy, **(B)** denoting laryngeal endoscopy, **(C)** denoting otoscopy, and **(D)** denoting auditory canal irrigation. The horizontal axis indicates the viral exposure level per second, or “viral RNA load/s”.

We evaluated the effectiveness of various intervention approaches by analyzing the effective filtration rates of N95 masks (94.10%), medical surgical masks (51.90%), and simple cotton masks (38.10%). This study indicates that wearing an N95 mask can effectively prevent airborne transmission at close range, reducing the risk of infection to 2.44%. The risk of infection can be decreased to 14.81% when wearing a medical surgical mask and to 36.05% with a basic cotton mask (see Figure 10).

## Discussion

Respiratory infectious diseases (RIDs) have become increasingly common and diverse in recent years (27). Accurately forecasting infections caused by these diseases is crucial, necessitating the development of more sophisticated prediction algorithms to analyze the complex patterns associated with them (28). In contrast, traditional methods for predicting respiratory infectious disorders may fail to identify illnesses promptly and accurately (29). Therefore, to implement effective prevention and control strategies for the public in addressing the challenges posed by respiratory infectious diseases (RIDs), ongoing, in-depth research in the field of disease prediction is essential.

To enhance early disease surveillance and address the limitations of existing respiratory infectious disease prediction models, this study assessed the risks associated with the SARS-CoV-2 virus using computational fluid dynamics (CFD) techniques. The findings indicated that the maximum risk of infection during medical procedures was 42.72%, while the average viral exposure per second in outpatient departments was 0.012564 viral RNA loads. These results were consistent with those reported by Zhang et al. (30). The heightened risk of infection in outpatient environments can be attributed to close contact, which is identified as one of the six principal factors facilitating the transmission of respiratory infections (31, 32). Observations during various medical procedures, such as laryngoscopy, nasal endoscopy, otoscopy, and external auditory canal irrigation, revealed that the interpersonal distance between patients and healthcare providers was frequently less than 1.5 meters, with the closest recorded distance being as minimal as 0.39 meters. Considering that airborne aerosols and droplet particles can travel at least 1.5 meters under standard environmental conditions (33), the results of this study suggest that the average distance maintained between patients and healthcare providers was merely 0.62 meters. This situation aligns with the World Health Organization’s definition of close contact (34), thereby fostering an environment particularly conducive to the transmission of respiratory infectious diseases (RIDs). This study also found that the relative facial position of HCWs during close contact with patients significantly influenced the degree of viral exposure. The highest level of viral exposure, reaching up to 0.033007 viral RNA loads per second, was observed during face-to-face interactions between HCWs and patients, as determined by investigations utilizing computational fluid dynamics (CFD) simulations. When patients and HCWs interacted face-to-side, the viral exposure measured 0.007670 viral RNA loads per second. In contrast, when positioned back-to-back, the viral exposure was negligible. These findings are consistent with those of Nielsen et al. (35). Following an analysis of 161,917 behavioral data points, we found that during medical procedures, the predominant facial orientation between patients and healthcare professionals was face-to-face (30.5%), followed by face-to-side (17.1%). When considered together, excessively close interpersonal distance and specific facial orientation may be the primary contributors to the elevated risk of respiratory infectious disease transmission in outpatient departments (42.27%). However, we often overlook potential micro-level influencing factors in our daily work practices; for example, we tend to neglect subtle variations in facial orientation and interpersonal distance. Additionally, the complexity of the standardization process and the challenges associated with collecting behavioral data limit our ability to observe micro-level behaviors. Drawing on existing research, this paper proposes a novel approach to gathering behavioral data by integrating semi-supervised machine learning algorithms with machine vision technologies. This innovative approach has the potential to generate new opportunities for predicting respiratory infections. More importantly, we believe that interventions focusing on facial orientation and interpersonal distance are more relevant to the preventive and control needs of the post-epidemic era compared to other strategies. Therefore, we recommend that medical personnel in outpatient ENT departments adhere strictly to the use of masks and face shields during relevant procedures, while also minimizing unnecessary face-to-face interactions and close contact. This is particularly critical during outbreaks of novel infectious diseases or influenza, as compliance with appropriate behaviors and protective measures is essential for ensuring the health and safety of both healthcare providers and patients.

In conclusion, this study developed a micro-level risk assessment model for respiratory infectious diseases (RIDs) by utilizing behavioral data from patients and healthcare staff. Moreover, this model also provides a novel perspective on the management and prevention of respiratory infectious diseases (RIDs). Although this study differs from previous models designed to predict the risk of respiratory infectious diseases (RIDs), it complements them and will enhance the prognosis, prevention, and management of respiratory infectious diseases (RIDs).

This study has the following limitations. First, this research only considered the risk of infection to healthcare workers from airborne transmission of the virus, while touching contaminated surfaces is also an important route for virus transmission. Second, this study used semi-supervised machine learning algorithms to process behavioral data, so the collected behavioral data may still differ from the actual situation. Finally, the behavioral data in this study came from a single center with a limited sample size, so the data characteristics may not represent other departments in the Beijing area. Therefore, the next phase of research needs to expand the scope of the study to obtain a wide and diverse data sample, thereby more accurately assessing the risk of transmission of respiratory infectious diseases.

## Data Availability

The raw data supporting the conclusions of this article will be made available by the authors, without undue reservation.
